# Discrimination of Multi-Origin Chinese Herbal Medicines Using Gas Chromatography-Mass Spectrometry-Based Fatty Acid Profiling

**DOI:** 10.3390/molecules181215329

**Published:** 2013-12-11

**Authors:** Xiao-Jing Zhang, Jian-Feng Qiu, Lan-Ping Guo, Ying Wang, Peng Li, Feng-Qing Yang, Huanxing Su, Jian-Bo Wan

**Affiliations:** 1 State Key Laboratory of Quality Research in Chinese Medicine, Institute of Chinese Medical Sciences, University of Macau, Macao 999078, China; E-Mails: zhangxiaojingky@163.com (X.-J.Z.); jianfeng_qiu@hotmail.com (J.-F.Q.); pengli@umac.mo (P.L.); 2 Institute of Chinese Materia Medica, China Academy of Chinese Medical Sciences, Beijing 100700, China; E-Mail: soil2006@126.com; 3 School of Chemistry and Chemical Engineering, Chongqing University, Chongqing 400030, China; E-Mails: wyjenny0106@hotmail.com (Y.W.); ysfhqx@hotmail.com (F.-Q.Y.)

**Keywords:** fatty acid profiling, *Curcuma* species, multi-origin Chinese herbal medicine, gas chromatography-mass spectrometry, multivariate statistical analysis

## Abstract

Multi-origin Chinese herbal medicines, with herbs originating from more than one species of plants, is a common phenomenon but an important issue in Traditional Chinese Medicines (TCMs). In the present study, a gas chromatography-mass spectrometry (GC-MS)—based fatty acid profiling approach to rapidly discriminate multi-origin Chinese medicines in terms of species and medicinal parts was proposed and validated using tuberous roots (*Curcumae* Radix) and rhizomes (*Curcumae* Rhizoma and *Curcumae* Longae Rhizoma) derived from four *Curcuma* species (e.g., *C. wenyujin*, *C. kwangsiensis*, *C. phaeocaulis* and *C. longa*) as models. Both type and content of fatty acids varied among different species of either tuberous roots or rhizomes, indicating each species has its own fatty acid pattern. Orthogonal partial least squares discriminant analysis (OPLS-DA) and hierarchical clustering analysis (HCA) based on dataset of global fatty acid profiling showed that either tuberous roots or rhizomes samples could be clearly classified into four clusters according to their species. Furthermore, those tested samples could also be discriminated in terms of their medicinal parts (e.g., tuberous root and rhizome). Our findings suggest that the proposed GC-MS-based fatty acid profiling followed by multivariate statistical analysis provides a reliable platform to discriminate multi-origin Chinese herbal medicines according to species and medicinal parts, which will be helpful for ensuring their quality, safety and efficacy.

## 1. Introduction

One medicinal herb originated from more than one species of plants, and one plant used as two or more medicines in terms of their different parts, are very popular phenomena in Traditional Chinese Medicines (TCMs). According to the statistics in the *Chinese Pharmacopoeia* (2005 edition), a total of 142 TCMs are multi-origin, including 89 of two species, 42 of three species, and 11 of more than three species [1]. However, each species has its own hereditary characteristics and phenotype, as well as its specific way to adapt to external environment, such as temperature, rainfall, soil and time exposure to sun, leading to typical primary and secondary metabolite patterns. In the recent years, an increasing number of studies have demonstrated that the chemical profiles of multi-origin Chinese herbal medicines, including Epimedii folium [2], *Curcuma* rhizomes [3] and Flos lonicerae [4], are obviously disparate according to different species, although they are used as the same herb in the *Chinese Pharmacopoeia*. Thus, the use of multi-origin Chinese herbal medicines might greatly affect the stability and homogeneity of TCM quality, as well as the clinical efficacy and safety. Morphological characterization, including macroscopic and microscopic, is a conventional method to identify the origin of herb [5]. Recently, molecular genetic identification has been also used to authorize the species of Chinese medicines [6]. However, these methods are tedious, time-consuming and experience-based. Therefore, the development of a simple and effective approach to discriminate the different species of multi-origin Chinese herbal medicines is of utmost importance for the quality control and clinical application of TCMs.

Fatty acids are considered as energy sources and structural components of the cell membrane. In the past decade, fatty acid profiling has been extensively applied in the discrimination of the healthy controls from several diseases, such as nonalcoholic steatohepatitis [7], type II diabetes mellitus [8], Alzheimer disease [9] and chemically induced liver injury [10], as well as in the identification of microbial species [11,12]. Furthermore, our previous study demonstrated that fatty acid characteristics could clearly discriminate three *Panax* species [13]. Therefore, we hypothesized that fatty acid profiling might be used to discriminate multi-origin Chinese herbal medicines according to their species, which is based on the fact that each species of plant with unique genotype presents the various metabolites, including fatty acid profiling [14]. To test the hypothesis, two typical multi-origin Chinese herbal medicines, including *Curcumae* Radix and *Curcumae* Rhizoma, were chosen as the model herbs.

The genus *Curcuma*, belonging to the family Zingiberaceae, includes about 80 accepted species of rhizomatous plants distributed around the world. About 20 *Curcuma* species occur in China, of which a few have been used as TCMs and/or food supplements for a long time [6]. According to the records in the *Chinese Pharmacopoeia* (2010 edition), *Curcumae* Radix (“*Yujin*” in Chinese) is the dry tuberous roots of four *Curcuma* species, including *C. wenyujin* Y. H. Chen et C. Ling, *C. kwangsiensis* S. G. Lee et C. F. Liang, *C. phaeocaulis* Val. and *C. longa* L. [5]. It is commonly used in the treatment of hepatitis, cholecystitis, hyperlipidaemia and cancer [15]. Interestingly, the different parts of the plants derived from aforementioned four *Curcuma* species are also used for TCM which have diverse therapeutic indications. The rhizomes of three *Curcuma* species, including *C. wenyujin*, *C. kwangsiensis* and *C. phaeocaulis* are used as “*Ezhu*”, which possesses the anti-cancer and anti-viral activities [1]. The rhizome of *C. longa* is commonly used as “*Jianghuang*”, which exhibits multiple pharmacological activities, including anti-oxidation, anti-atherosclerosis, anti-depression and immune activation [16,17]. Although belonging to the same genus, those four *Curcuma* species present great variation in chemical composition, which might lead to different pharmacological activities. Thus, the authentication of those medicinal herbs is very important to ensure their safety and efficacy. Due to their similar morphological characteristics, it is difficult to distinguish their origins of raw materials, either derived from root or rhizome. In the past years, a few methods, such as gas chromatography-mass spectrometry (GC-MS) [18], high-performance liquid chromatography (HPLC) [3], twice development thin layer chromatography (TLC) [19], capillary electrophoresis (CE) [20], and GC-MS-based metabolomics [21], have been developed to discriminate the different species of *Curcuma* samples according to their chemical diversity of specific secondary metabolites, especially sesquiterpenes. As primary metabolites, fatty acids present in almost all plants, which makes it the much wider application prospect. In the present study, using tuberous roots and rhizomes of four *Curcuma* species as two model herbal medicines, a simple GC-MS based fatty acid profiling method was proposed to rapidly discriminate the different species of multi-origin Chinese herbal medicines. 

## 2. Results and Discussion

### 2.1. Validation of the GC-MS Method

The robustness or ruggedness of analytical method, including instrumental analysis and sample preparation, should be evaluated to guarantee statistical difference is not derived from analytical drift in a chemometric study. The developed GC-MS method has been validated by using precision, stability and reproducibility tests (Table 1). An intra-day precision was achieved by analyzing the fatty acid methyl esters (FAMEs) mixed standards for six times successively, and 11 fatty acids detected in tested sample were selected to evaluate the instrumental drift. Overall, the content variation of fatty acids was less than 3.2%, respectively, suggesting excellent instrumental performance during whole analytical run. Due to oxidative susceptibility of fatty acids, particularly polyunsaturated fatty acid (PUFA), the stability of methylated fatty acids should be tested. A freshly prepared *C. kwangsiensis* rhizome sample (EW-2) was analyzed at different time intervals of 0, 2, 4, 6, 8 and 10 h. As results, FAMEs derived from the tested sample were stable for at least 10 h at ambient room temperature with overall variation of 0.8%–6.1%. In addition, to test the repeatability, sample of EW-2 was also divided into six and parallelly prepared under the methylation conditions, and then analyzed by GC-MS. The repeatability of each fatty acid in the tested sample was less than 8.9%. In conclusion, the developed GC-MS method was robust with good precision, stability and repeatability.

**Table 1:** Stability, repeatability and precision data of the investigated fatty acids.

FA	Precision		Stability		Repeatability	
	Content (%)	RSD (%)	Content (%)	RSD (%)	Content (%)	RSD (%)
C15:0	9.16 ± 0.22	2.39	0.57 ± 0.02	2.95	0.57 ± 0.03	4.49
C16:0	9.69 ± 0.15	1.52	25.01 ± 0.21	0.84	25.53 ± 0.32	1.27
C17:0	9.85 ± 0.07	0.68	0.61 ± 0.01	1.95	0.58 ± 0.03	5.15
C18:0	9.97 ± 0.05	0.46	4.23 ± 0.06	1.52	4.45 ± 0.39	8.77
C18:1 n-9	10.14 ± 0.02	0.23	7.59 ± 0.08	1.07	7.71 ± 0.12	1.58
C18:2 n-6	9.74 ± 0.04	0.43	39.39 ± 0.31	0.77	38.71 ± 0.46	1.18
C18:3 n-3	9.46 ± 0.02	0.26	17.3 ± 0.59	3.4	17.03 ± 0.35	2.08
C20:0	8.71 ± 0.08	0.86	1.54 ± 0.05	3.11	1.58 ± 0.08	4.92
C20:1	10.87 ± 0.10	0.96	0.55 ± 0.02	2.77	0.52 ± 0.05	8.90
C22:0	6.74 ± 0.22	3.24	1.17 ± 0.07	6.01	1.21 ± 0.04	3.7
C24:0	5.66 ± 0.10	1.69	2.02 ± 0.11	5.38	2.09 ± 0.10	4.96

### 2.2. Fatty Acid Composition of Tuberous Roots and Rhizomes Derived from Four Curcuma Species

**Figure 1:**
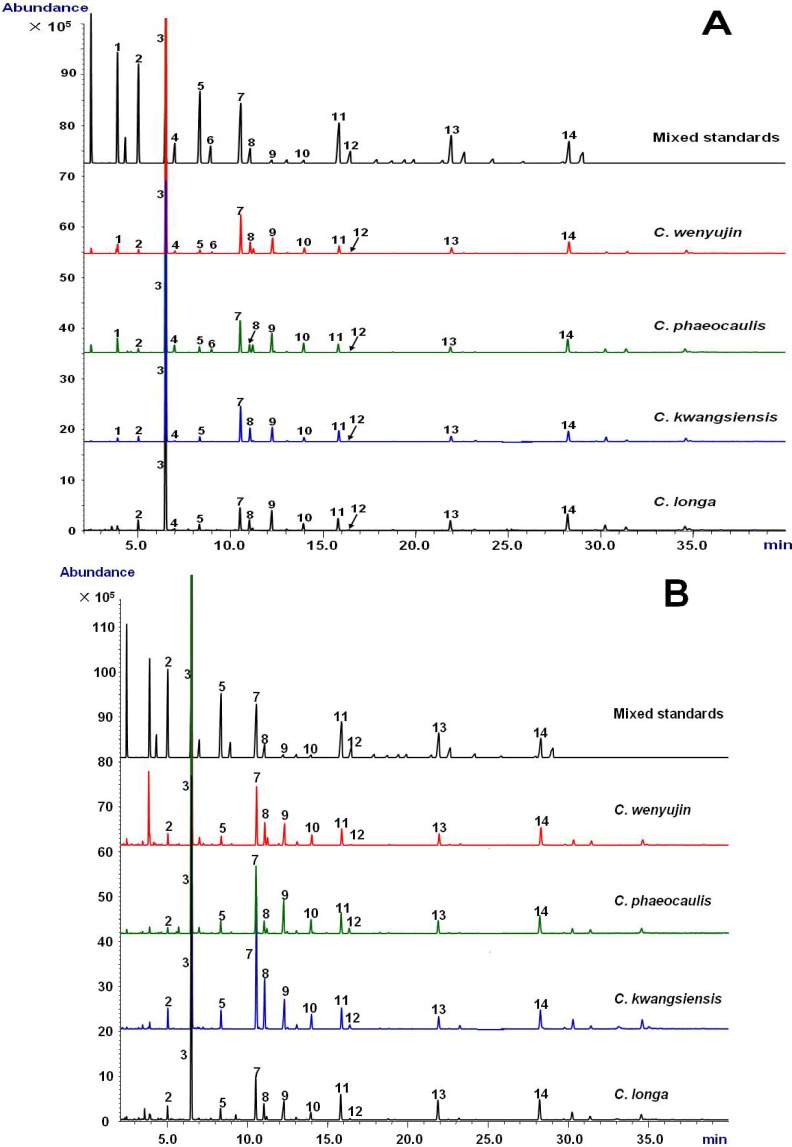
Representative extracted ion chromatograms of *m*/*z* 74 of mixed standards and methyl ester of fatty acids in tuberous roots (**A**) and rhizomes (**B**) derived from *C. wenyujin*, *C. phaeocaulis*, *C. kwangsiensis* and *C. longa*. The mixed standards contain 32 fatty acid methyl esters as described in the Chemicals section. Fatty acids were represented as the corresponding methyl esters. **1**. C14:0; **2**. C15:0; **3**. C16:0; **4**. C16:1 n-7; **5**. C17:0; **6**. C17:1 n-7; **7**. C18:0; **8**. C18:1 n-9; **9**. C18:2 n-6; **10**. C18:3 n-3; **11**. C20:0; **12**. C20:1 n-9; **13**. C22:0; **14**. C24:0.

The validated GC-MS method was employed to determine fatty acid compositions in tuberous roots and rhizomes derived from four *Curcuma* species. Typical extracted ion chromatograms (EICs) of *m*/*z* 74 were shown in Figure 1. The peaks corresponding to fatty acids were well separated on an Omegawax 250 column within 30 min. A total of 14 fatty acids in methyl ester form, including eight saturated fatty acids (SFAs, e.g., C14:0, C15:0, C16:0, C17:0, C18:0, C20:0, C22:0 and C24:0), four monounsaturated fatty acids (MUFAs, e.g., C16:1 n-7, C17:1 n-7, C18:1 n-9, and C20:1 n-9) and two PUFAs (e.g., C18:2 n-6 and C18:3 n-3), were observed in tuberous root of *C. wenyujin* and *C. phaeocaulis*. However, C17:1 n-7 was not detected in tuberous root of *C. kwangsiensis* and *C. longa*. C14:0 was also not found in tuberous root of *C. longa*. Only 11 fatty acids, excluding C14:0, C16:1 n-7 and C17:1 n-7, were determined consistently in rhizomes from *C. wenyujin*, *C. kwangsiensis* and *C. longa*.

*Curcuma* species herbs are rich in the volatile oils and there are many undesired volatile oil peaks in total ion chromatogram (TIC) of GC-MS, which severely interfered with the quantification of fatty acids (data not shown). In order to avoid the interference of overlapping peaks and make the method simple and easy to deal with, EIC of ion *m*/*z* 74 was used to quantify fatty acids in *Curcuma* samples. By normalizing each TIC peak area calculated by EIC of ion *m*/*z* 74 as percentage of total fatty acids, the relative contents of investigated fatty acids in *Curcuma* samples were calculated and summarized in Table 2. In all *Curcuma* samples, either tuberous roots or rhizomes, total content of PUFA was accounted for a relatively high proportion (more than 50%), sequentially followed by total SFA and MUFA. Although palmitic acid (C16:0), linoleic acid (C18:2 n-6) and α-linolenic acid (C18:3 n-3) were main fatty acid compositions in all species, their proportion were extensively distinct in term of *Curcuma* species, especially in tuberous roots. *C. longa* possessed the highest relative content of C18:2 n-6, while C16:0 and C18:3 n-3 were more abundant in *C. phaeocaulis*. The contents of minor fatty acids, e.g., C15:0, C16:1 n-7, C18:0, C18:1 n-9, *etc*., as well as the ratio of n-6/n-3 PUFA also varied greatly among different species of either tuberous roots or rhizomes. Taken together, it is suggested that each species has its own fatty acid pattern. Even so, it was difficult to discriminate different species of tuberous roots and rhizomes by visual observation of the fatty acid profiles detected by GC-MS, as major types of fatty acid among species were similar.

### 2.3. Multivariate Statistical Analysis

**Figure 2:**
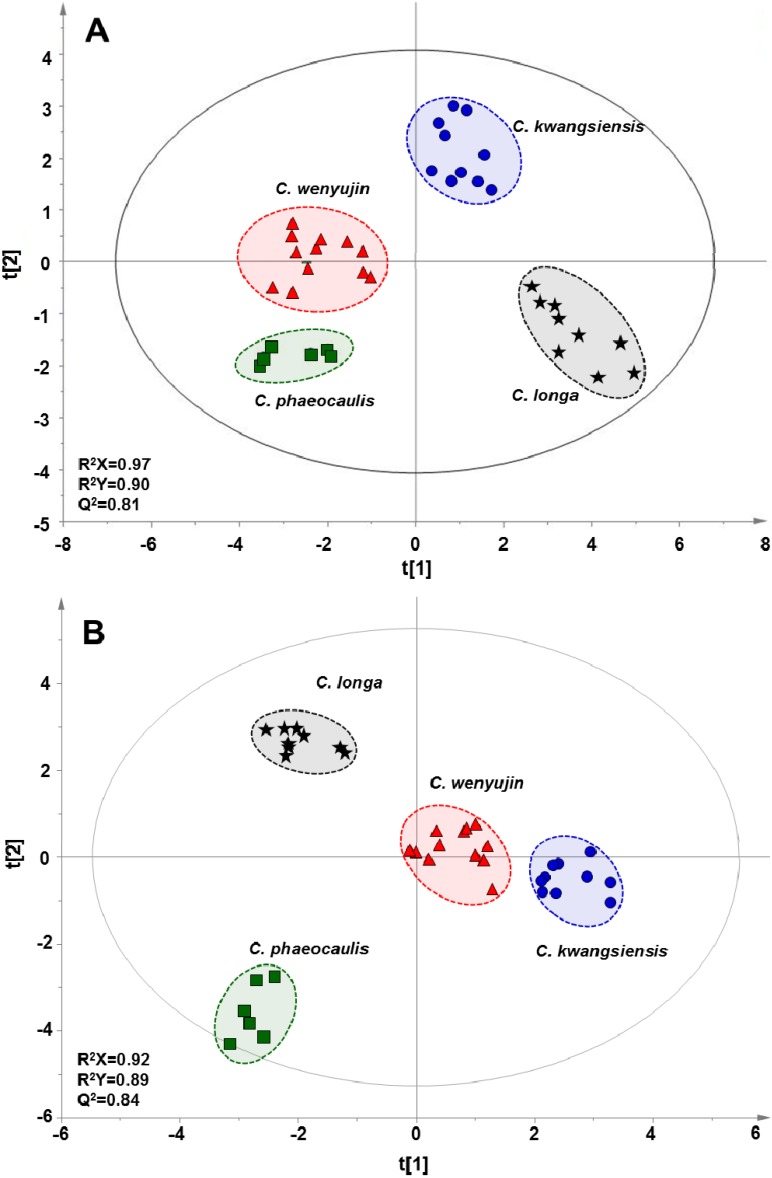
OPLS-DA score plots based on fatty acids profiles of (**A**) tuberous roots and (**B**) rhizomes derived from *C. wenyujin*, *C. phaeocaulis*, *C. kwangsiensis* and *C. longa*.

**Figure 3:**
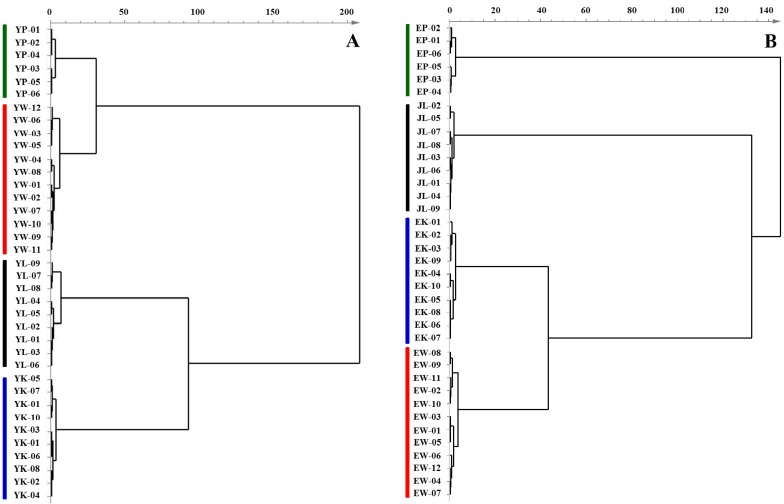
HCA dendrograms resulting from the contents of fatty acids in (**A**) tuberous roots and (**B**) rhizomes derived from *C. wenyujin*, *C. phaeocaulis*, *C. kwangsiensis* and *C. longa*.

With the datasets of the contents of fatty acids in tuberous roots and rhizomes, orthogonal projections to latent structure discriminant analysis (OPLS-DA), a supervised statistical modeling method for pattern recognition, was separately applied to discriminate four *Curcuma* species based on their differences in fatty acid profiles. After unit variance (UV) and mean-centering, all data were represented as scores in a coordinate system of latent variables. As shown in Figure 2, all tested samples, either tuberous roots or rhizomes, were clearly classified into four regions in terms of species, e.g., *C. wenyujin*, *C. kwangsiensis*, *C. phaeocaulis* and *C. longa*, in the scores plots according to the differences in their global fatty acid profiles. Three parameters, including R^2^X, R^2^Y, and Q^2^, are usually used to evaluate the quality and reliability of OPLS-DA model. Generally, their values close to 1.0 indicate an excellent fitness for the model, and the values of R^2^ and Q^2^ should be differ less than 0.3 [22,23]. In present score plots, all observations, except one rhizome sample from *C. phaeocaulis*, fell within the Hotelling T2 (0.95) ellipse, where the model fit parameters were 0.97 of R^2^X, 0.90 of R^2^Y, and 0.81 of Q^2^ using the content of fatty acids in tuberous roots samples as variations (Figure 2A), and 0.92 of R^2^X, 0.89 of R^2^Y, and 0.84 of Q^2^ for rhizomes samples (Figure 2B), which suggested that the constructed OPLS-DA model has the excellent fitness and predictive capability. In addition, in order to visualize similarities among samples through linkage distances, hierarchical cluster analysis (HCA), an unsupervised learning method, was employed to generate dendrograms according to fatty acid profiles of 37 batches of tuberous roots and rhizomes, respectively. A very efficient method, named Ward, was applied as measurement to analyze variances among clusters. The correlations of each *Curcuma* sample were expressed by the linkage distances in HCA dendrograms. As shown in Figure 3, either tuberous roots or rhizomes samples could be unambiguously divided into four main clusters according to their species. These results suggested that based on their fatty acid profiling, the *Curcuma* species of both tuberous roots and rhizomes could be discriminated according to their species using GC-MS analysis and multivariate statistical analysis, such as OPLS-DA and HCA. It is most plausible that each species has its own hereditary characteristics or genotype, leading to unique fatty acid pattern. Definitely, the environmental stimuli or growth place could also affect the fatty acid profiling, however, this kind of influence is extremely limited. As shown in Figure 2 and Figure 3, the same species collected from different region could not be separated by OPLS-DA and HCA analysis.

**Table 2:** Fatty acid profiles of the tuberous roots and rhizomes from four *Curcuma* species.

Peak No.	Fatty Acid (%)		Tuberous Root				Rhizome			
	Common Name	Symbol	CW ( *n* = 12)	CP ( *n* = 6)	CK ( *n* = 10)	CL ( *n* = 9)	CW ( *n* = 12)	CP( *n* = 6)	CK ( *n* = 10)	CL ( *n* = 9)
1	Tetradecanoic acid	C14:0	0.66 ± 0.31 ^a^	0.83 ± 0.19 ^a^	0.32 ± 0.12 ^b^	ND	ND	ND	ND	ND
2	Pentadecanoic acid	C15:0	0.32 ± 0.06 ^a^	0.28 ± 0.02 ^b^	0.46 ± 0.05 ^c^	0.76 ± 0.09 ^d^	0.56 ± 0.05 ^a^	0.26 ± 0.02 ^b^	0.68 ± 0.12 ^c^	0.77 ± 0.08 ^d^
3	Palmitic acid	C16:0	28.32 ± 1.10 ^a^	29.40 ± 0.96 ^b^	27.09 ± 1.5 ^c^	24.55 ± 0.54 ^d^	25.45 ± 1.10 ^a^	25.82 ± 0.38 ^a^	25.55 ± 1.89 ^a^	22.88 ± 0.64 ^b^
4	Palmitoleic acid	C16:1 n-7	2.85 ± 2.25 ^a^	3.62 ± 0.18 ^a^	0.58 ± 0.24 ^b^	0.53 ± 0.18 ^b^	ND	2.01 ± 0.35	ND	ND
5	Heptadecanoic acid	C17:0	0.33 ± 0.04 ^a^	0.47 ± 0.04 ^b^	0.52 ± 0.07 ^b^	0.69 ± 0.11 ^c^	0.62 ± 0.07 ^a^	0.63 ± 0.05 ^a^	0.85 ± 0.16 ^b^	0.77 ± 0.05 ^b^
6	Heptadecenoic acid	C17:1 n-7	0.62 ± 0.41 ^a^	1.55 ± 0.28 ^b^	ND	ND	ND	0.85 ± 0.36	ND	ND
7	Stearic acid	C18:0	4.43 ± 0.88 ^a^	3.11 ± 0.76 ^b^	4.28 ± 0.59 ^a^	3.08 ± 0.43 ^b^	4.78 ± 0.58 ^a^	3.79 ± 0.17 ^b^	5.64 ± 0.53 ^c^	3.39 ± 0.17 ^d^
8	Oleic acid	C18:1 n-9	5.49 ± 0.84 ^a^	3.02 ± 0.49 ^b^	10.78 ± 2.4 ^c^	4.57 ± 0.56 ^d^	8.92 ± 0.89 ^a^	4.08 ± 0.47 ^b^	10.83 ± 1.51 ^c^	6.28 ± 1.28 ^d^
9	Linoleic acid	C18:2 n-6	37.29 ± 2.35 ^a^	35.66 ± 1.19 ^a^	40.10 ± 0.79 ^b^	42.78 ± 1.15 ^c^	39.73 ± 0.92 ^a^	38.26 ± 1.18 ^b^	38.61 ± 2.36 ^a^^,b^	42.61 ± 1.09 ^c^
10	α-linolenic acid	C18:3 n-3	15.52 ± 0.93 ^a^	17.65 ± 1.0 ^b^	10.56 ± 2.62 ^c^	16.25 ± 0.71 ^d^	14.57 ± 1.47 ^a^	17.59 ± 0.65 ^b^	13.37 ± 2.42 ^a^	15.69 ± 1.20 ^c^
11	Arachidic acid	C20:0	0.90 ± 0.17 ^a^	0.99 ± 0.21 ^a^	1.34 ± 0.58 ^a^	1.68 ± 0.23 ^b^	1.33 ± 0.21 ^a^	1.64 ± 0.21 ^b^	0.91 ± 0.32 ^c^	2.20 ± 0.37 ^d^
12	Eicosenoic acid	C20:1 n-9	0.26 ± 0.06 ^a^	0.37 ± 0.13 ^a^^,b^	0.47 ± 0.12 ^b^	0.46 ± 0.06 ^c^	0.54 ± 0.08 ^a^	1.85 ± 0.08 ^b^	1.02 ± 0.58 ^c^	0.56 ± 0.10 ^a^
13	Docosanoic acid	C22:0	0.81 ± 0.20 ^a^	0.86 ± 0.09 ^a^^,b^	1.06 ± 0.33 ^b^	1.63 ± 0.22 ^c^	1.18 ± 0.18 ^a^	1.34 ± 0.26 ^b^	0.81 ± 0.27 ^c^	2.14 ± 0.41 ^d^
14	Lignoceric acid	C24:0	2.27 ± 0.40 ^a^	2.19 ± 0.21 ^a^	2.44 ± 0.73 ^a^	3.03 ± 0.36 ^b^	2.33 ± 0.39 ^a^	1.88 ± 0.67 ^b^	1.73 ± 0.51 ^b^	2.73 ± 0.46 ^c^
SFA			37.95 ± 1.36 ^a^	38.12 ± 1.26 ^a^	37.50 ± 0.81 ^a^	35.41 ± 0.81 ^b^	36.24 ± 1.34 ^a^	35.37 ± 0.74	36.18 ± 2.29	34.89 ± 1.12 ^b^
MUFA			9.21 ± 2.22 ^a^	8.58 ± 1.10 ^a^	11.83 ± 2.55 ^b^	5.56 ± 0.57 ^c^	9.45 ± 0.91 ^a^	8.79 ± 0.94 ^a^	11.85 ± 1.94 ^c^	6.81 ± 1.27 ^b^
PUFA			52.79 ± 2.73 ^a^	53.30 ± 1.61 ^a^	50.66 ± 2.57 ^b^	59.03 ± 0.96 ^c^	54.31 ± 1.45 ^a^	55.85 ± 1.23 ^b^	51.980 ± 1.27 ^c^	58.30 ± 1.15 ^d^
n-6/n-3			2.41 ± 0.18 ^a^	2.0 3 ± 0.14 ^b^	4.05 ± 1.14 ^c^	2.64 ± 0.16 ^d^	2.75 ± 0.29 ^a^	2.18 ± 0.11 ^b^	2.99 ± 0.64 ^a^	2.73 ± 0.25 ^a^

Average followed by different letters are significantly different (*p* < 0.05). ND, not detected; SFA, saturated fatty acids; MUFA, monounsaturated fatty acids; PUFA, polyunsaturated fatty acids; CW, *C. wenyujin*; CP, *C. phaeocaulis*; CK, *C. kwangsiensis*; CL, *C. longa*.

**Figure 4:**
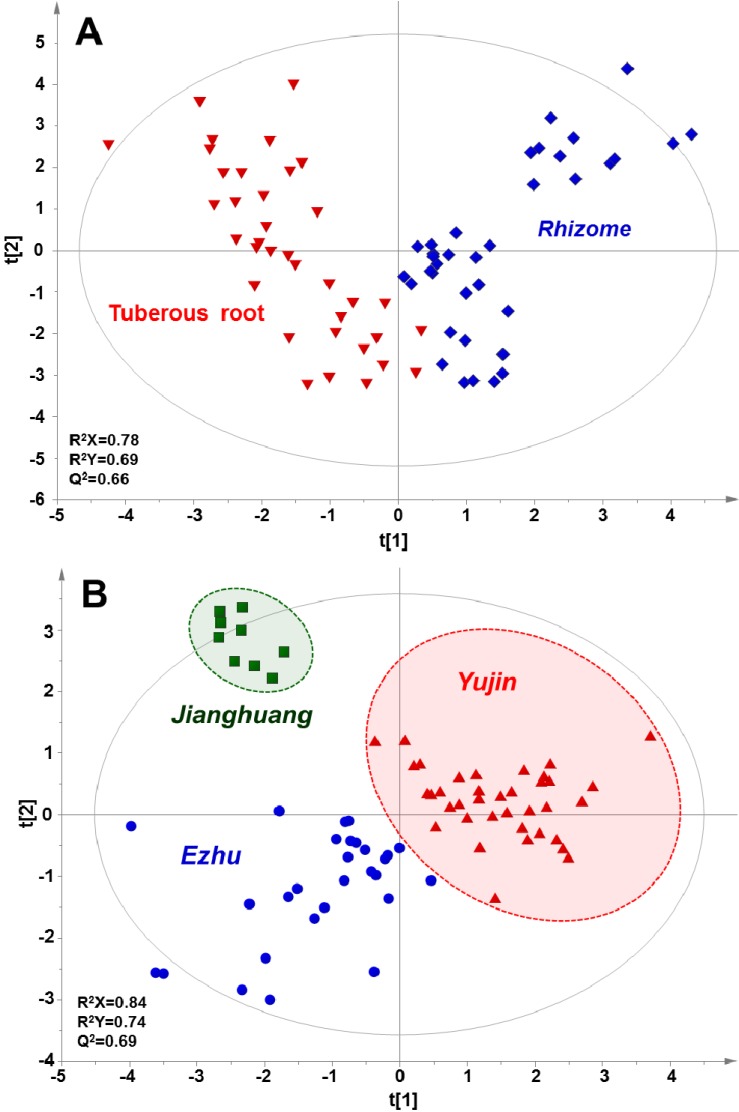
OPLS-DA score plots based on fatty acids profiles of 74 batches of *Curcuma* samples, including tuberous roots and rhizomes. (**A**) Samples were defined as tuberous roots and rhizomes, (**B**) Samples were defined as “*Yujin*”, “*Ezhu*” and “*Jianghuang*”. The rhizomes of three *Curcuma* species, including *C. wenyujin*, *C. kwangsiensis* and *C. phaeocaulis* are used as “*Ezhu*”. The rhizome of *C. longa* is commonly used as “*Jianghuang*”, and the tuberous roots of aforementioned four *Curcuma* species were defined as “*Yujin*”.

In order to further strength the potential discriminative capability of fatty acid profiling, the fatty acid datasets of tuberous roots and rhizomes were combined and then subjected to OPLS-DA model. After redefining the classes, tuberous roots and rhizomes samples derived from four *Curcuma* species were mainly divided into two clusters in score plot with values of R^2^Y and Q^2^ of 0.69 and 0.66 respectively (Figure 4A). In addition, we re-defined the samples as those three Chinese medicines, e.g., “*Yujin*”, “*Ezhu*” and “*Jianghuang*”. As shown in Figure 4B, these three herbs could be unambiguously distinguished in the score plot of OPLS-DA with Q^2^ value of 0.69. These results suggested that the different parts, e.g., tuberous root and rhizome, originated from four *Curcuma* species, as well as three *Curcuma*-based Chinese medicines could be sufficiently discriminated by fatty acid profiling.

In the present study, we demonstrated that fatty acid profiling could clearly distinguish four *Curcuma* species of either tuberous roots or rhizomes using GC-MS followed by multivariate statistical analysis. Compared to the conventional morphological identification or chromatographic fingerprints or bioactive component determination method, this proposed approach is simple and flexible. More importantly, unlike specific secondary metabolites, fatty acids, as primary metabolites, could be found in almost all plants, which make it in much wider application. Our findings suggest that the GC-MS based fatty acid profiling provided reliable discrimination of multi-origin Chinese herbal medicines in terms of species and medicinal parts, which will be helpful for ensuring their safety and efficacy.

## 3. Experimental

### 3.1. Herbal Materials and Chemicals

A total of 37 batches of *Curcuma* plant materials, including tuberous roots and their rhizomes, were collected from the agricultural farms in Zhejiang (*C. wenyujin*), Guangxi (*C. kwangsiensis*), and Sichuan Province (*C. phaeocaulis* and *C. longa*), China, during December 2012. The botanical origins of the *Curcuma* plants in forms of whole plants were identified morphologically during the field collection. All tuberous root and rhizome samples were dried in an oven at 60 °C. *Curcumae* Radix samples (*Yujin*) included the tuberous roots from 12 batches of *C. wenyujin* (YW-1-YW-12), 10 batches of *C. kwangsiensis* (YK-1-YK-10), six batches of *C. phaeocaulis* (YP-1-YP-6) and 9 batches of *C. longa* (YL-1-YL-9). Correspondingly, *Curcumae* Rhizoma samples (*Ezhu*) included the rhizomes from 12 batches of *C. wenyujin* (EW-1-EW-12), 10 batches of *C. kwangsiensis* (EK-1-EK-10) and six batches of *C. phaeocaulis* (EP-1-EP-6), and *Curcumae* Longae Rhizoma (*Jianghuang*) included the rhizomes from nine batches of *C. longa* (JL-1-JL-9). The voucher specimens of those tuberous roots (*Curcumae* Radix) and rhizomes (*Curcumae* Rhizoma and *Curcumae* Longae Rhizoma) from four *Curcuma* species were deposited at the Institute of Chinese Medical Sciences, University of Macau, Macao, China.

HPLC-grade methanol, *n*-hexane and boron trifluoride (BF_3_) in methanol (14%) were purchased from Sigma-Aldrich (St. Louis, MO, USA). GLC-461 reference standard, consisting of 32 FAMEs, was purchased from Nu-Chek Prep (Elysian, MN, USA), which specifically included as follows: 13 SFAs (e.g., C4:0, C6:0, C8:0, C10:0, C12:0, C14:0, C15:0, C16:0, C17:0, C18:0, C20:0, C22:0 and C24:0), 7 MUFAs (e.g., C14:1 n-9, C16:1 n-7, C17:1 n-7, C18:1 n-9, C20:1 n-9, C22:1 n-9 and C24:1 n-9), and 12 PUFAs (e.g., C18:2 n-6, C18:3 n-6, C18:3 n-3, C20:2 n-6, C20:3 n-6, C20:3 n-3, C20:4 n-6, C20:5 n-3, C22:2 n-6, C22:4 n-6, C22:5 n-3 and C22:6 n-3). Deionized water was prepared by a Milli-Q purification system (Millipore, Bedford, MA, USA).

### 3.2. Sample Preparation

Sample preparation of *Curcuma* samples was conducted according to previous reports [24,25] with some modification. Briefly, samples were pulverized and accurately weighted (approximately 50 mg) for methyl esterification reaction. After transferred to a glass screw-cap tube, samples were mixed with hexane (1.5 mL) and 14% BF_3_/methanol solution (1.5 mL). Subsequently, the mixture was blanketed with nitrogen and heated at 100 °C in a MK200-2 dry bath incubator (AoSheng, Hangzhou, China) for 1 h. Methyl esters were extracted in hexane phase after the addition of 1 mL H_2_O and then centrifuged for 5 min at 1,000 g. The upper hexane layer was removed and concentrated under liquid nitrogen gas, and the residue was redissolved in hexane (200 µL) and subsequently subjected to GC-MS analysis.

### 3.3. GC-MS Analysis

Fatty acid methyl esters were analyzed by using an Agilent GC-MS system (Agilent Technologies, Palo Alto, CA, USA) consisting of an Agilent 6890 gas chromatography and an Agilent 5973 mass spectrometer. Separation was achieved on an Omegawax™ 250 fused silica capillary column (30 m × 0.25 mm i.d., 0.25 µm film thickness, Supelco, Bellefonte, PA, USA) under the optimized oven temperature program: initial temperature set at 180 °C and held for 3 min; ramped to 240 °C at 2 °C/min, and then held at 240 °C for 7 min. Overall, the total run time was 40 min. The injection volume was 2 µL with a split ratio of 1:15, and the injector temperature was set at 250 °C. High-purity helium (>0.9999) was used as carrier gas at a flow rate of 1.5 mL/min. The mass spectrometer was operated in electron-impact (EI) mode at ionization energy of 70 eV. The spectra were acquired in the *m*/*z* range of 35 to 550 between 2 min to 40 min with the scan rate of 0.34 s per scan. The temperatures of quadrupole and ionization source were set at 150 °C and 280 °C, respectively.

Fatty acids were identified in forms of their methyl esters, mainly based on their chromatographic and mass spectral characteristics. MS Search 2.0 database, developed by National Institute of Standards and Technology (NIST), was searched to rigorously assign potential structures for all peaks detected in TIC. FAMEs were also confirmed by comparing mass spectra and retention times with those of the mixed reference standards eluted under identical chromatographic conditions. Because fragment ion (*m*/*z* 74) presents in all detected FAMEs, EIC of ion *m*/*z* 74 was employed for quantification of fatty acids in order to avoid the interference of overlapping peaks. TIC peak area of each FAME was calculated by multiplying its EIC peak area by corresponding coefficient (TIC peak area of reference standard divided by its EIC peak area). The relative contents of fatty acids in samples were calculated by normalization of the obtained TIC peak areas as the percentages of total fatty acids.

### 3.4. Data Processing

All data were expressed as mean ± standard deviation (SD). Statistical differences in the content of each fatty acid among tuberous root and rhizome samples derived from four *Curcuma* species was assessed by one-way analysis of variance (ANOVA) using SPSS 19.0 (SPSS, Inc., Chicago, IL, USA) after verifying normal distribution of dependent variables by Kolmogorov-Smirnov test. A *p* value < 0.05 was considered significant.

The normalized data set of tuberous roots (*Curcumae* Radix) and rhizomes (*Curcumae* Rhizoma and *Curcumae* Longae Rhizoma) were separately imported into to SIMCA-P version 13.0 (Umetrics, Umeå, Sweden) for multivariate pattern recognition analysis. All samples were subjected to visual classification using OPLS-DA and HCA, according to their intergroup difference and similarity in fatty acid profiles, respectively. After UV scaling and mean-centering, OPLS-DA, a method with ability to effectively filter unrelated variations under supervised model, was carried out to examine the distributions and discriminations among groups according to the difference in fatty acid pattern. OPLS-DA model were evaluated and interpreted in terms of R^2^X (cum), R^2^Y(cum) and Q^2^(cum) in score plot. The parameters of R^2^X and R^2^Y represent explanatory capacity on variables in X and Y matrices, while Q^2^ suggests the predictive capability of the model. The values of R^2^ and Q^2^ close to 1.0 indicate an excellent fitness for the method [26,27]. In addition, in order to evaluate correlation of tested samples, HCA was used to generate the dendrogram using linkages and distances among them based on their fatty acids characteristics. A method named Ward, a very efficient method for analysis of variance between clusters, was chosen as measurement.

## 4. Conclusions

In the present study, we demonstrated that fatty acid profiling could clearly distinguish four *Curcuma* species of either tuberous roots or rhizomes using GC-MS followed by multivariate statistical analysis. Compared to the conventional morphological identification or chromatographic fingerprint or bioactive component determination method, this proposed approach is simple and flexible. More importantly, unlike specific secondary metabolites, fatty acids, as primary metabolites, could be found in almost all plants, which make it in much wider application. Our findings suggest that the GC-MS based fatty acid profiling provides reliable discrimination of multi-origin Chinese herbal medicines in terms of species and medicinal parts, which will be helpful for ensuring their safety and efficacy.
